# ASO Author Reflections: Towards Patient-Tailored Management of Extremity Soft Tissue Sarcoma

**DOI:** 10.1245/s10434-021-10040-y

**Published:** 2021-04-17

**Authors:** I. Acem, M. A. J. van de Sande, C. Verhoef

**Affiliations:** 1 Surgical Oncology and Gastrointestinal Surgery, Erasmus MC Cancer Institute, Rotterdam, Netherlands; 2 Department of Orthopedic surgery, Leiden University Medical Center, Leiden, Netherlands

## Past

A multimodality approach is often used in the management of high-grade soft tissue sarcoma of the extremity (eSTS). Limb-sparing surgery is the keystone of eSTS management, often accompanied with radiotherapy (RTX) and/or chemotherapy (CTX). The National Comprehensive Cancer Network (NCCN) and European Society for Medical Oncology (ESMO) guideline recommend RTX and CTX in selected groups of high-risk patients.^1^,^2^ However, the identification of these high-risk patients who benefit from perioperative treatment remains challenging. In the last decade, several prognostic tools and prognostic gene expression profiles have been developed to facilitate clinical decision-making for the indication of multimodality therapy. This marks a shift from a one-size-fits-all approach to a patient-tailored approach for the management and surveillance of eSTS.

## Present

Our cross-sectional study aimed to acquire insight into the variation of eSTS management across the world and to assess the importance of several clinicopathologic risk factors for the indication of multimodality therapy.^3^ Although studies have shown better patient outcomes when adhering to the clinical guidelines, this study showed remarkable variation in eSTS management and surveillance across continents and physician specialties. Notably, a wide variation in attitude towards perioperative CTX was found across specialties. Mainly surgical and orthopedic oncologists did not feel there is sufficient evidence for the use of perioperative CTX in high-grade eSTS. Furthermore, Physicians were predominantly positive about the use of prognostic tools for the indication of multimodality therapy. In addition, this study showed a wide variation in the frequency of follow-up and follow-up modality, with a remarkable difference in preference for chest computed tomography (CT) scan versus chest X-ray across continents.

## Future

The optimal management of patients with eSTS remains an important field of research. Recent developments of prediction tools and advances in genomics and proteomics may allow for better patient selection for multimodality therapy. Because in recent years some retrospective studies have been performed to assess the use of these tools for the indication of multimodality therapy, further studies are needed to improve patient selection for the indication of multimodality therapy, with a special focus on perioperative CTX and new perioperative modalities. Also, the cost-effectiveness of chest CT scan versus chest X-ray and the use of prognostic tools for patient-tailored follow-up regimen should be explored. Given the rarity and heterogeneity of eSTS, large collaborative efforts of international sarcoma centers are crucial to further improve patient-tailored management of eSTS.
